# Genome Analysis of the First Extensively Drug-Resistant (XDR) *Mycobacterium tuberculosis* in Malaysia Provides Insights into the Genetic Basis of Its Biology and Drug Resistance

**DOI:** 10.1371/journal.pone.0131694

**Published:** 2015-06-25

**Authors:** Chee Sian Kuan, Chai Ling Chan, Su Mei Yew, Yue Fen Toh, Jia-Shiun Khoo, Jennifer Chong, Kok Wei Lee, Yung-Chie Tan, Wai-Yan Yee, Yun Fong Ngeow, Kee Peng Ng

**Affiliations:** 1 Department of Medical Microbiology, Faculty of Medicine, University of Malaya, Kuala Lumpur, Malaysia; 2 Codon Genomics SB, Seri Kembangan, Selangor Darul Ehsan, Malaysia; University of Padova, Medical School, ITALY

## Abstract

The outbreak of extensively drug-resistant tuberculosis (XDR-TB) has become an increasing problem in many TB-burdened countries. The underlying drug resistance mechanisms, including the genetic variation favored by selective pressure in the resistant population, are partially understood. Recently, the first case of XDR-TB was reported in Malaysia. However, the detailed genotype family and mechanisms of the formation of multiple drugs resistance are unknown. We sequenced the whole genome of the UM 1072388579 strain with a 2-kb insert-size library and combined with that from previously sequenced 500-bp-insert paired-end reads to produce an improved sequence with maximal sequencing coverage across the genome. *In silico* spoligotyping and phylogenetic analyses demonstrated that UM 1072388579 strain belongs to an ancestral-like, non-Beijing clade of East Asia lineage. This is supported by the presence of a number of lineage-specific markers, including *fadD28*, *embA*, *nuoD* and *pks7*. Polymorphism analysis showed that the drug-susceptibility profile is correlated with the pattern of resistance mutations. Mutations in drug-efflux pumps and the cell wall biogenesis pathway such as *mmpL*, *pks* and *fadD* genes may play an important role in survival and adaptation of this strain to its surrounding environment. In this work, fifty-seven putative promoter SNPs were identified. Among them, we identified a novel SNP located at -4 T allele of *TetR/acrR* promoter as an informative marker to recognize strains of East Asian lineage. Our work indicates that the UM 1072388579 harbors both classical and uncommon SNPs that allow it to escape from inhibition by many antibiotics. This study provides a strong foundation to dissect the biology and underlying resistance mechanisms of the first reported XDR *M*. *tuberculosis* in Malaysia.

## Introduction

The global rise of extensively drug-resistant tuberculosis (XDR-TB) has jeopardized efforts in the treatment and control of TB. XDR-TB is commonly caused by *Mycobacterium tuberculosis* which is resistant to rifampicin, isoniazid, fluoroquinolones, and one of the three second-line injectable antibiotics: kanamycin, amikacin or capreomycin [1]. XDR-TB cases have since been reported by 100 countries worldwide, including South Africa [2], India [3], East Asia [4, 5] and European countries [6–8]. In most XDR-TB-burdened countries, previous attempts to address this epidemic have been obviously unsuccessful as they face huge obstacles in the rapid diagnosis and control of this drug-resistant TB. In Malaysia, we have also recently reported, to our knowledge, the first XDR-TB case [9]. However, little is known about the nature of this strain and such information is obviously required for better diagnosis and control of this drug-resistant TB.

The evolutionary origin and nature of the XDR *M*. *tuberculosis* strains remain unknown. Two possible explanations have been postulated to describe the occurrence and transmission of XDR-TB worldwide. The first explanation involves clonal expansion and spreading of one strain harboring several drug-resistance mutations [10–12]. Alternatively, it appears that drug-resistance mutational events occurred multiple times separately in different strains, proposing repeated selection from a pool of pre-existing strains through the chemotherapeutic usage pattern rather than the spread of a single drug-resistant strain [13]. The advent of high throughput Next Generation Sequencing technologies (NGS) provide a step forward in drug-resistant TB research to decipher the biology, adaptation and evolution of these “extreme” strains. Although there are whole genome sequence data for XDR in the international databases [12, 14–18], the whole genome sequences of XDR strains from Southeast Asia tropical countries are still lacking. In 2013, we sequenced the genome of the first XDR *M*. *tuberculosis* strain UM 1072388579 with a 500-bp insert-size library [9]. Here, we sequenced the UM 1072388579 genome with a 2-kb insert-size library and combined the genomic sequence data with that from the previous sequencing using the small DNA insert library to improve genome assembly. In Malaysia, only one XDR-TB case has been reported so far. The origin and the genotype of XDR *M*. *tuberculosis* in the country are totally unclear. In this study, we demonstrated that the UM 1072388579 strain harbors an ancestral-like spoligotype, which is close to the Beijing clade of East Asia lineage.

At this stage of knowledge, there is still a need for thorough investigation of SNPs present in drug resistance associated genes and intergenic regions (IGRs) although several classical mutations such as *katG*, *ndh*, *pncA*, *rpoB* and *gyrA* have been reported [19]. With this goal in mind, we comprehensively analyzed the underlying molecular genetic basis for drug resistance in order to identify novel resistance mutations. Genetic characterization of clinical isolates requires systematic SNP analysis. Thus, it is hoped that the thoroughly analysis of the UM 1072388579 genome will help to provide critical information for the XDR *M*. *tuberculosis* in Malaysia.

## Materials and Methods

### Ethic statement

The isolate used in this study was obtained from the previously published study [9]. All labels on the source sample have been erased with the exception of sample type and clinical diagnosis. Thus there is no information traceable back to the patient from whom the isolate was obtained. As such, this genomic study is exempt from ethical approval in our teaching hospital. (http://www.ummc.edu.my/view/content.php?ID=VGxSWlBRPT0=).

### Strain and drug-susceptibility testing

*M*. *tuberculosis* strain UM 1072388579 was isolated from sputum of a 57 year-old man in the Mycobacteriology Laboratory, University Malaya Medical Centre, Kuala Lumpur, Malaysia. Culture of the clinical isolate was performed using a BACTEC MGIT 960 Culture system (Becton Dickinson) as described in the previous study [20]. An hundred microliter aliquot of MGIT broth was removed for microscopy examination with Auramine and Ziehl-Neelsen staining to ensure the presence of acid fast bacilli on the day of detection of culture positive. Gram-staining was performed on the same aliquot of broth to confirm there was no bacterial contamination. For identification of mycobacterial species, 1 mL of aliquot of MGIT broth was transferred to a screw-capped 1 mL tube for GenoType Mycobacterium CM assay [20].

Drug susceptibility testing of the clinical isolate was performed on the isolated *M*. *tuberculosis* strain using a BACTEC MGIT 960 Culture system [20]. The concentrations of drugs used for testing were 1.0 μg/mL rifampicin, 0.2 μg/mL isoniazid, 2.5 μg/mL kanamycin, 1.0 μg/mL amikacin, 2.5 μg/mL capreomycin, 2.0 μg/mL streptomycin, 1.0 μg/mL ciprofloxacin, 2.0 μg/mL ofloxacin, 5.0 μg/mL ethambutol, 5.0 μg/mL ethionamide and 4.0 μg/mL para-salicylic acid. The UM 1072388579 isolate was tested using the MTBDR*plus* and MTBDR*sl* assays but the commercially available tests would not be able to identify resistance to specific drugs due to the lack of well-known mutations associated to a drug resistant phenotype.

### Genomic DNA extraction

Genomic DNA was carried out in a Class III biological safety cabinet according to the chemical lysis method [21]. In this study, a hundred Lowenstein-Jensen culture slants was used to obtain sufficient total DNA (40 μg) for both small and large insert library sizes whole genome sequencing. All visible colonies from Lowenstein-Jensen medium were collected into a microcentrifuge tube containing 350 μL Tris-EDTA buffer (10 mM Tris-base and 1 mM EDTA, pH 8.0). The suspension was mixed with 50 μL of lysozyme (10 mg/mL) and 10 μL of RNAse A and incubated at 37°C for 2 hours. The mixture was then incubated with 50 μL of proteinase K (10 mg/mL) at 55°C for 20 minutes. Next, the mixture was mixed with 100 μL of 10% (w/v) of SDS and incubated at 37°C for 1 hour. After 1 hour incubation, proteins were precipitated with 5 M NaCl and 100 μL of Cetyltrimethyl Ammonium Bromide (CTAB), followed by incubation at 65°C for 10 minutes. Genomic DNA was then purified and precipitated with equal volume of chloroform: isoamyl alchohol (24:1, v/v) and isopropanol, respectively. The tube was then centrifuged at 10,000 ×*g* for 20 minutes at 4°C. It is important to pipette the DNA very slowly when transferring the aqueous (upper) phase to avoid disturbing the material at the interface. Genomic DNA was washed from the pellet by adding 650 μL of ice-cold 70% (v/v) ethanol and centrifuged at 10,000 ×*g* for 5 minutes at 4°C. The washing step was repeated for three times. Finally, the pellet was dissolved in nuclease-free distilled water. The pellet of DNA was not dried completely as desiccated DNA is very hard to dissolve. The quantity and quality of the extracted genomic DNA was determined using NanoDrop 2000c spectrophotometer (Thermo Fisher Scientific). Pure DNA sample is indicated by A260/A280 ratio between 1.8–2.0. In addition, a total sample quantity of 6 μg and 40 μg is required for short-insert library and large-insert library, respectively.

### Genome sequencing and assembly

Whole genome sequencing of UM 1072388579 was performed using Illumina HiSeq 2000 Sequencer (Illumina) in a 2×90 bp paired-end mode on 500-bp and 2-kb library sizes. Illumina library was prepared using TruSeq v3 Reagent Kits (Illumina). Qualified DNA sample was sheared into smaller fragments by Covaris S/E210. The selected library fragments were purified through gel-electrophoresis, then selectively enriched and amplified by PCR. All raw reads were first pre-processed using FASTX-Toolkit (http://hannonlab.cshl.edu/fastx_toolkit/) trimming bases with a Phred quality below Qv20 from the 3’-end of the reads, retaining small-insert reads ≥ 80 bp and large-insert reads ≥ 30 bp. Two nucleotides were trimmed from the 5’-terminal of the reads. All reads with 40% bases having Qv ≤ 20 were then filtered out. Pre-processed reads from both libraries were assembled with Velvet version 1.2.07 [22] using k-mer setting = 63, insert length = 503, -ins_length_sd = 103 and -min_pair_count = 15. Additional parameter of-shortMatePaired = yes was set for large insert library. The generated sequences assembled from the Velvet assembly were then scaffolded using SSPACE Basic v2.0 [23] with more stringent parameters than software default to achieve higher accuracy assembly (parameters:-z 100, -k 15, -a 0.3, -n 30 and -T 10). GapFiller v1.10 was employed to perform gap filling by using paired-end sequencing data from both libraries [24, 25] with parameter settings-m = 60, -o = 15, -r = 0.8,–n = 30, -t = 30 and -T = 10.

### Gene prediction and annotation

Protein coding sequences of UM 1072388579 were predicted using GeneMarkS v4.10d [26]. Annotation of coding sequences for UM 1072388579 was completed using BLAST (Basic Local Alignment Search Tool) searches against the NCBI non-redunctant (nr), SwissProt and COG databases. rRNAs and tRNAs were identified using RNAmmer v1.2 [27] and tRNAscan-SE v1.3.1 [28], respectively.

### Determination of Principal genotypic group

To determine the principal genotypic group (PGG), the coding sequences of katG and gyrA were first retrieved from the sequenced genome using Artemis v12.0 sequence viewer [29]. The nucleotide polymorphism at katG codon 463 and gyrA codon 95 of UM 1072388579 was determined by compared with the katG and gyrA protein sequences retrieved from the H37Rv strain.

### *In silico* Spoligotyping

Spoligotype of UM 1072388579 was inferred *in silico* from raw sequence files (fastq format) using SpolPred software with default parameters [30]. The result was then matched to the SpolDB4 [31] and SITVITWEB [32] spoligotype databases to determine the spoligotype pattern.

### Genome-wide SNPs analysis

Single nucleotide polymorphism (SNP) discovery was performed using MUMmer v3.23 [33]. Nucmer algorithm (default parameters) of the MUMmer software package was used to align genomic sequences (contigs or complete genomes) to the H37Rv reference genome (RefSeq NC_000962) and primary SNP calls were generated by using show-snps algorithm (parameters:-Clr) from the same software package. SNPs from potential paralogous regions were excluded for further analysis. The SNPs discovered in UM 1072388579 were annotated based on the H37Rv genome annotation and classified as synonymous, non-synonymous or intergenic using ANNOVAR [34].

### Phylogenetic analysis

SNPs in 50 *Mycobacterium* genomes (including UM 1072388579, 48 *M*. *tuberculosis* representing the main six MTBC lineages, and the outgroup species *M*. *bovis*) (S1 Table) were identified using MUMmer v3.23. A total of 20,708 SNPs were found to be common in all the 50 genomes. SNPs in each genome were concatenated into single contiguous sequences and aligned. The resulting SNP-based alignment was used to perform Bayesian MCMC inference analysis using MrBayes v3.2.2 [35] (ran for 1,500,000 MCMC generations with sampling every 500 generations). The average SD of split frequencies was below 0.01 after 1.5 million generations, indicative of convergence. Posterior probabilities were averaged over the final 75% of trees (25% burn in). The phylogenetic tree was plotted using FigTree v1.4.2 (http://tree.bio.ed.ac.uk/software/figtree/).

### Identification of SNPs in promoter region

One kilobase (kb) of upstream regions of annotated genes was retrieved from the UM 1072388579 genome using Artemis v12.0 sequence viewer [29]. The promoters were then predicted from these non-coding sequences using the Neural Network Promoter Prediction computer program (parameters: type of organism: prokaryote; minimum promoter score: 0.8) [36]. The predicted promoter regions were cross-checked with the SNPs identified in UM 1072388579 using in-house Perl script. Briefly, the Perl script compared the genomic location of each SNP against the predicted promoter regions and identified putative SNPs residing in the putative promoters.

## Results and Discussion

### Characteristics of the UM 1072388579 isolate

*M*. *tuberculosis* strain UM 1072388579 was isolated from a 57-year-old patient in University Malaya Medical Centre. The isolate was sub-cultured and re-tested for the susceptibility to first-line and second-line drugs twice using BACTEC MGIT 960 Culture system, prior to whole genome sequencing. The drug susceptibility tests confirmed that UM 1072388579 isolate is XDR *M*. *tuberculosis*. It was resistant to rifampicin, isoniazid, streptomycin, ethambutol, pyrazinamide, kanamycin, amikacin, capreomycin, ethionamide and fluoroquinolones (ofloxacin and ciprofloxacin) but sensitive to para-salicylic acid.

### Genome sequence of UM 1072388579 strain from Malaysia

To gain insight into its underlying molecular mechanism in the development of XDR phenotype, we have sequenced the UM 1072388579 genome. The UM 1072388579 genome was sequenced to >99% completion with ~100-fold depth of genome sequence coverage. A total of 8,394,316 paired reads (0.73 Gb) of a 500-bp insert-size library and 11,190,552 paired reads (0.97 Gb) of a 2-kb insert-size library were generated by Illumina HiSeq 2000 Sequencing system. The mate pair combined sequencing data showed a lower number of contigs and a higher N50 value compared to that generated from the single short DNA insert library (Table 1). The assembly yielded a combined total length of 4,370,957 bases in 80 contigs (≥200 bases) which were then placed into 19 scaffolds (≥1,000 bases) with paired-end sequencing data from both libraries. The assembly has an N50 scaffold size of 1,081,051 bases with an average G+C content of 65.15%. The high G+C content indicates the more biased codon frequencies of the proteins in UM 1072388579 isolate [37]. A total of 4,159 coding DNA sequences (CDS) with length of ≥33 amino acids was predicted, from which 4,126 and 3,122 proteins were functional annotated using local BLAST similarity searches against NCBI nr and SwissProt databases, respectively (S2 Table). All the proteins were also ascribed to 21 different functional groups based on Clusters of Orthologous Groups (COGs) (S2 Table and S1 Fig). Apart from the poorly characterized categories: categories R (general functions prediction only) and S (function unknown), our result showed that UM 1072388579 strain has a great potential to synthesize various group of lipophilic molecules (palmitate, tuberculostearic acid and mycolic acid), essential amino acids, enzyme co-factors and vitamins (S2 Table and S1 Fig). By comparing the sequenced UM 1072388579 to H37Rv, we observed that the *moaB3* gene, which is involved in molybdopterin biosynthesis, is totally missing in our isolate. The biosynthesis of molybdopterin is involved in the physiology and intracellular survival of *M*. *tuberculosis* [38]. Therefore, the *moaB3* gene might not be important for pathogenesis. Since there are 21 genes dedicated to the molybdopterin biosynthesis [37], it is possible that other family members compensate for the loss of *moaB3* gene. Furthermore, our pipeline showed that there is one rRNA operon for 5S, 16S and 23S and 45 tRNAs in the genome (Table 1).

**Table 1:** Comparison of short-insert paired-end (500-bp) and mate pair combined sequencing data.

Details	Short-insert paired-end (500-bp)	Paired-end and mate pair combined (500-bp and 2-kb)
**Sequencing depth**	~100×	~100×
**Total length of sequences (bp)**	4,290,533	4,370,957
**Total number of contigs (≥200 bp)**	184	80
**Total number of scaffolds (≥1,000 bp)**	89	19
**N50 (bp)**	108,779	1,081,051
**G+C (%)**	65.40	65.15
**Number of predicted protein coding-gene (≥ 33 amino acids)**	4,099	4,151
**Annotated protein coding regions (nr)**	4,069	4,126
**tRNAs**	45	45
**5s rRNA**	1	1
**16s rRNA**	1	1
**23s rRNA**	1	1

We then captured all genetic differences of UM 1072388579 by comparing with the H37Rv reference genome. From that, a total of 1,397 genic SNPs were localized to the UM 1072388579. Of these polymorphisms, 536 and 861 of them were identified as synonymous SNPs (sSNPs) and non-synonymous SNPs (nsSNPs), respectively. The 861 nsSNPs were distributed in 644 genes. The details of identified SNPs in UM 1072388579 are available in the supplemental material (S3 Table).

### Genotype of UM 1072388579

Principal genetic group (PGG) of our clinical isolate was first defined based on the combination of nucleotide polymorphisms at katG codon 463 and gyrA codon 95 [39]. We found that UM 1072388579 is grouped into PGG1 with KatG463 CTG (Leu) and GyrA95 ACC (Thr), in contrast to PGG3 [KatG463 CGG (Arg) and GyrA95 AGC (Ser)] for H37Rv strain. PGG1 organisms are evolutionarily old and related to *Mycobacterium bovis*, notorious for causing bovine tuberculosis [39]. Sreevatsan et al. (1997) also reported that PGG1 organisms are ancestral to PGG2 and PGG3 *M*. *tuberculosis*. Based on the PGG grouping, UM 1072388579 is thought to be an ancient strain, although this evolutionary hypothesis that was proposed by Sreevatsan et al. (1997) is based on only two nsSNPs [39].

Six major lineages of *M*. *tuberculosis* have been described, including the Indo-Oceanic (East African Indian, EAI), East Asian (non-Beijing and Beijing), East African-Indian (Central Asian, CAS), Euro-American (Haarlem, Latin American Mediterranean (LAM), T, X, Urganda and Ural clades), West African 1 and West African 2 lineages [31]. XDR-TB has been associated with almost every *M*. *tuberculosis* genotype family, such as Euro-American (Haarlem, LAM, T, X, Ural) [12, 14, 40], Central Asian (CAS) [41] and Beijing [40, 42]. Here, we determined the spoligotype of UM 1072388579 by using SpolPred and matching the read to the SpolDB4 database. The result showed that UM 1072388579 strain exhibits an almost complete, ancestral-like spoligotype pattern (777777777777731, ST. no 246) [43, 44] as it misses spacer 40 only (Table 2). However, the result showed that it cannot be clustered unambiguously in any known genotype family (Table 2). Non-Beijing isolates with such ancestral-like spoligotype patterns belong to the East Asia lineage [43, 45].

**Table 2:** Spoligotypes of UM 1072388579 and FJ05194 strains.

Strain	SIT^a^	Clade
UM 1072388579	246	Unassignable
FJ05194	643	Unassignable
LN130	1	Beijing

^a^ SIT correspond to the Spoldb4 international database code accessible at http://www.pasteur-guadeloupe.fr/tb/bd_myco.html.

A SNP in *fadD28* gene (codon 507) was previously used as a marker to identify *M*. *tuberculosis* isolates of the Beijing clade and genetically similar non-Beijing East Asia lineage isolates [46]. Conversely, no SNP was observed in *fadD28* for isolates from other genotype families (EAI, T, LAM, MANU, Haarlem and S clades) [46]. To support that UM 1072388579 strain belongs to the non-Beijing clade of East Asian lineage, we have identified lineage-specific SNPs at *fadD28* (I507I), *embA* (A2856G), *nuoD* (A201T) and *pks7* (C5787T) [47] in our isolate (Table 3).

**Table 3:** Lineage-specific SNPs shared between the UM 1072388579 and FJ05194.

Gene name	Lineage or sub-lineage	Lineage name	Nucleotidevariation	Amino acids variation	Reference
*fadD28*	2	East-Asian	C1521T	I507I	[46]
*embA*	2.1	East-Asian (non-Beijing)	A2856G	Q952Q	[47]
*nuoD*	2.1	East-Asian (non-Beijing)	A201T	E67D	[47]
*pks7*	2.1	East-Asian (non-Beijing)	C5787T	G1929G	[47]

**Fig 1:**
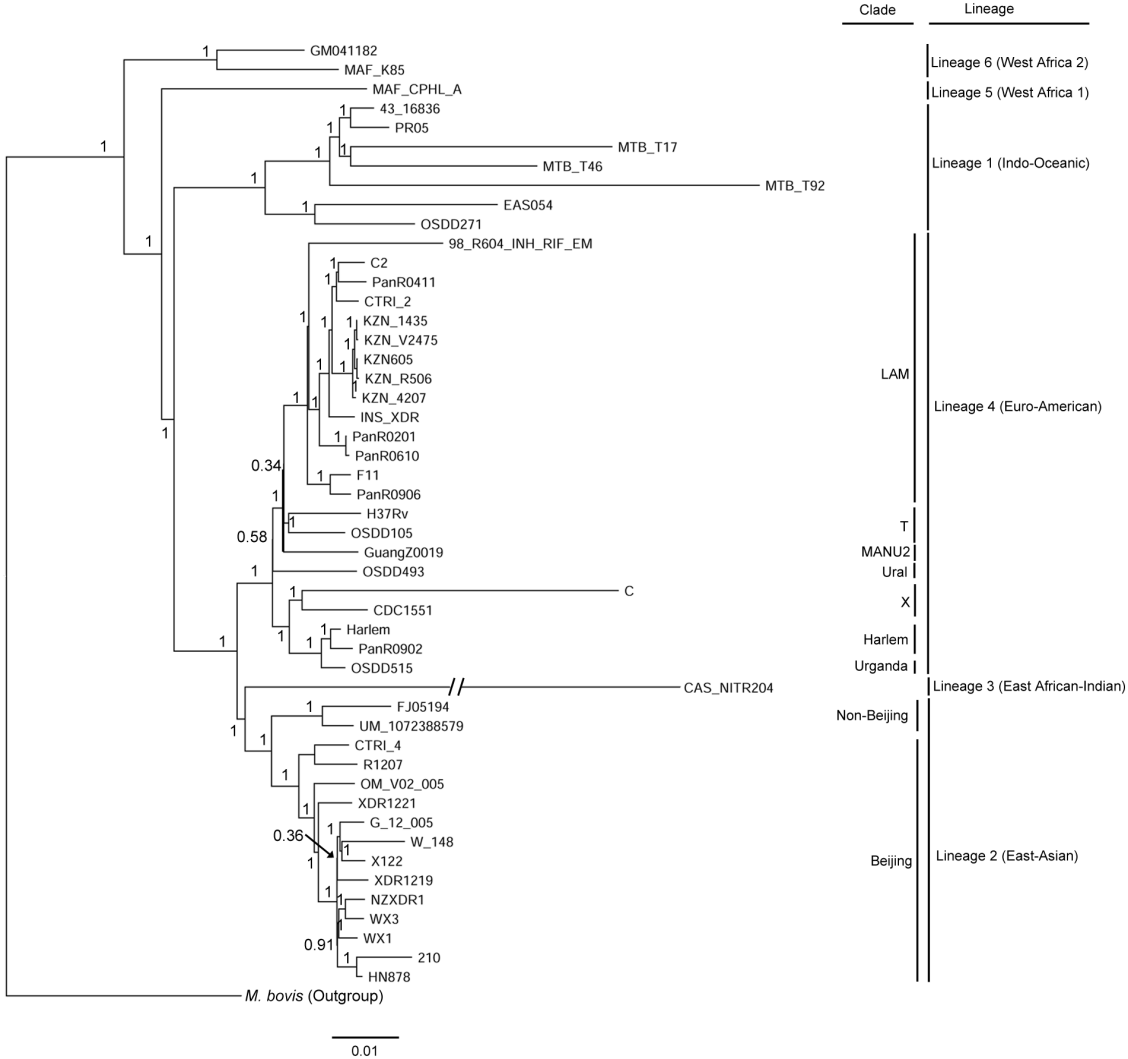
Comparative phylogenetic analysis of UM 1072388579 strain along with 50 previously published genomes. Phylogenetic tree was constructed based on overall SNPs extracted from genome sequences. The numbers on branches indicate Bayesian posterior probability values. *M*. *tuberculosis* isolates are clustered into respective genotype families based on spoligotyping-defined *M*. *tuberculosis* clade and lineage. The tree is rooted with *M*. *Bovis* BCG strain ATCC 35743 as outgroup.

A phylogenetic tree was then constructed based on a genome-wide set of SNPs to further gain insight into the genotype of UM 1072388579. Forty-nine additional published *M*. *tuberculosis* whole genomes were used for the comparative phylogenetic analysis (S1 Table). The result agrees well with a previously published phylogenetic tree [42, 48] and our isolate is clustered together with FJ05194, which is very close to the Beijing clade of East Asian lineage (Fig 1). FJ05194 strain was isolated from a patient in Fujian, China [15]. Both strains share similar lineage-specific markers [46, 47] that characterize the non-Beijing clade of East Asian lineage (Table 3). Considering that both strains of UM 1072388579 and FJ05194 were clustered together, we used the same algorithm to identify the spoligotype pattern of FJ05194. The result obviously showed that FJ05194 has an ancestral-like spoligotype pattern (777777757777771, ST. no 643) which is almost similar to our strain (Table 2). LN130 strain [40] with the known Beijing clade was included in the same analysis to confirm that the results are valid (Table 2). Non-Beijing isolates of ST no. 246 and 623 ancestral-like spoligotypes were reported to be genetically similar to Beijing clade [46], which have emerged from the South part of China, Guangxi autonomous region [44]. Wan and collaborators also proposed that such ancestral strains are mainly found in Guangxi, which are thought to be representatives of East Asia lineage branching out prior the occurrence of the modern Beijing isolates [44]. Overall, it is tempting to speculate that UM 1072388579 represents the ancestor of the Beijing clade that originated from the South part of China.

### Drug resistance patterns of UM 1072388579

We focused on the nsSNPs to determine the pattern of resistance mutations in each drug tested. Our polymorphism analysis demonstrated that the presence of resistance mutations were consistent with the drug-resistant phenotype (Table 4).

**Table 4:** Polymorphisms in UM 1072388579 strain inferred to be associated with drug resistance.

Drug resistance effect	Gene name	Mutation	Function
**Isoniazid**	*katG* (*Rv1908c*)	R463L and L141F	Catalase/peroxidase
**Rifampicin**	*rpoB* (*Rv0667*)	S450L	RNA polymerase (β subunit)
**Ethambutol**	*embB* (*Rv3795*)	Q497R	Arabinosyl transferase
**Ethionamide**	*ethA* (*Rv3854c*)	Ins862C	Monooxygenase
	*Rv0565c*	R110H	Probable monooxygenase
	*Rv1936*	Q68H	Probable monooxygenase
	*Rv3618*	D117Y	Probable monooxygenase
**Fluoroquinolone (ofloxacin and ciprofloxacin)**	*gyrA* (*Rv0006*)	E21Q, S91P, S95T and G668D	DNA gyrase (subunit A)
**Streptomycin**	*gidB* (*Rv3919c*)	G30V	7-methylguanosine methyltransferase
**Kanamycin and amikacin**	*eis* (*Rv2416*)	A22G	Enhanced intracellular survival protein
**Capreomycin**	*tlyA* (*Rv1694*)	Ins363GC	2’-O-methyltransferase
**Cycloserine**	*cycA* (*Rv1704c*)	R93L	D-serine/alanine/glycine transporter protein
	*DdlA* (*Rv2981*)	T365A	D-alanine:D-alanine ligase
	*alr* (*Rv3423*)	S22L	D-alanine racemase

The underlying molecular mechanisms of isoniazid resistance are complex as they are mediated by several genes including *katG*, *inhA*, *mabA*-*inhA* IGR and other unknown genes [49]. For UM 1072388579, isoniazid resistance might be associated with the R463L and L141F mutations in *katG*, the catalase/peroxidase that converts pro-drug isoniazid into an active form [50]. As previously reported [51, 52], R463L mutation has higher catalase activity than other resistance mutations and this may play a role in virulence of drug-resistant *M*. *tuberculosis* strains. However, R463L mutation is known not to be associated with isoniazid resistance [53]. L141F is a rare mutation in *katG*, which was frequently found in isolates with low level resistance to isoniazid [54], but it has not been confirmed to be associated to a resistant phenotype. Interestingly, previous study revealed that the catalase and isoniazid oxidation activities were not detected in KatG^L141F-R463L^ mutant [52]. In our case, it could be that a gene mutation conferring a lower level of resistance occurred first, followed by the acquisition of a second mutation in another gene that yielded a greater resistance for the strain. Collectively, they could have a cumulative effect in the development of isoniazid resistance in UM 1072388579.

With respect to rifampicin resistance, UM 1072388579 has the mutation of S450L in *rpoB* (β subunit of RNA polymerase). In addition, we identified two non-frameshift insertions (Ins629PP and Ins631S) in *ponA1* (penicillin-binding protein 1A) that is responsible for the biosynthesis of peptidoglycan layer in the cell wall. Previous functional genetic analysis confirmed that *M*. *tuberculosis* isolates containing the *ponA1* mutation had a greater survival advantage in the presence of rifampicin [55]. Previous studies showed that depletion of *ponA1* increase susceptibility to β-lactam antibiotics in *M*. *smegmatis* [56], suggested mutation in *ponA1* might play a role to inhibit activities of some antibiotics. However, the exact role of the *ponA1* mutations in UM 1072388579 remains unknown.

Ethambutol resistance of our isolate is most likely caused by the Q497R mutation in the transmembrane protein embB [57]. Interestingly, *embB* mutations have been reported that did not associate with ethambutol-resistance alone, but were related to both ethambutol and rifampicin resistance [58]. In UM 1072388579 isolate, the mutation in *embB* might have a role in the inhibition of both rifampicin and ethambutol activities.

In *M*. *tuberculosis*, DNA gyrase (encoded by *gyrA* and *gyrB* genes) is the common target for fluoroquinolones [59]. Polymorphism analysis shows a mutation in *gyrA* at the position of S91P. Ethionamide resistance in UM 1072388579 can be explained by the frameshift insertion in *ethA*. Like many anti-TB drugs, ethionamide is a pro-drug which requires metabolic activation by the enzyme ethA, a monooxygenase. Thus, a frameshift mutation in amino acid 288 caused by 1 bp insertion causes our clinical isolate to be resistant to ethionamide. We have also checked out SNPs in known genes coding the probable monooxygenase and found that UM 1072388579 strain carried mutations in *Rv0565c*, *Rv1936* and *Rv3618* (Table 4). The role of the mutations in these putative monooxygenase genes in fluoroquinolones resistance thus deserves greater attention.

The classical mutations that are associated with streptomycin resistance in *rpsL* (ribosomal protein S12) and in *rrs* (16S ribosomal RNA) genes were not identified in UM 1072388579. Previous study showed that mutations of these two genes contributed to only 70% cases of total streptomycin resistant isolates [60], implying that there must be other gene(s) which can be involved in the streptomycin resistance in UM 1072388579. Therefore, resistance to streptomycin is most likely attributed to the mutation in *gidB* at the position of G30V (Table 4). The *gidB* encodes SAM-dependent methyltransferase that catalyzes methylation at the position G527 in the 16S ribosomal RNA, giving an additional hydrophobic binding site for streptomycin [61]. As previously noted [61], mutations in *gidB* are the source for conferring low-level streptomycin resistance in *Streptomyces coelicolor*. Feuerriegel et al. (2012) reported that L16R, A205A and V110V in *gidB* were associated to phylogenetic features rather than being involved in drug resistance [62]. However, mutations at the position of G34A, V65G, G71A, V88G, L91P, S100F, A138V and A200E might be associated with streptomycin resistance as these mutations occur exclusively in streptomycin resistant strains [62]. Like our study, Spies et al. (2011) showed some mutations (codons 30, 45, 48, 49, 51, 52, 67, 75, 117 and 164) were associated with low-level streptomycin resistance in streptomycin resistant strains that contained no mutations in the *rpsL* or *rrs* genes [63]. Up until now, reasons for streptomycin-resistant strains exhibiting no mutation in *rrs* or *rpsL* and shift to mutation in *gidB* remain unknown [63, 64].

The drug-susceptibility test also showed that UM 1072388579 is resistant to kanamycin, amikacin and capreomycin. Unlike a previous study [65], kanamycin and amikacin resistance in UM 1072388579 might be associated with a non-synonymous mutation in *eis* gene (A22G). Capreomycin resistance of our isolate might be correlated with the frameshift insertion in *tlyA* (2’-O-methyltransferase). The functionality *tlyA* gene was disrupted by insertion mutation to catalyze methylation reaction on 23S rRNA (at nucleotide C1920) and 16S rRNA (at nucleotide C1409). Capreomycin resistance is attributed by the defect of this methylation reaction on 23S rRNA and 16S rRNA [66]. As in our work, a recent study discovered a novel frameshift insertion, Ins49GC in capreomycin-resistant *M*. *tuberculosis* strains [67].

It should be noted that our clinical isolate also harbors several mutations which are associated with D-cycloserine (Table 4), though susceptibility to these drugs was not examined. To date, the resistance mechanisms of D-cycloserine are poorly understood. D-cycloserine is a cyclic analog of D-alanine that hinders the action of D-alanine:D-alanine ligase (Ddl) and D-alanine racemase (Alr), which are involved in the peptidoglycan biosynthesis [19]. It is a second-line drug used to treat MDR-TB and XDR-TB because of less D-cycloserine-resistant *M*. *tuberculosis* is reported [68, 69]. However, it is less potent as compared to that of first-line drugs [69]. A recent comparative genomic analysis revealed that *M*. *bovis* bacillus Calmette-Guérin (BCG) containing a nsSNP in the *cycA* gene (bacterial D-alanine/D-serine/glycine transporter) is more resistant to D-cycloserine as compared to wild-type *M*. *tuberculosis* and *M*. *bovis* [70]. Additionally, we also identified non-synonymous mutations in *ddlA* (D-alanine:D-alanine ligase) and *alr* (D-alanine racemase) (Table 4). The mutations observed in *ddlA* and *alr* putatively prevent D-cycloserine from hindering with peptidoglycan biosynthesis. Taken together, mutations of *cycA*, *ddlA* and *alr* in UM 1072388579 are presumably involved in D-cycloserine resistance by blocking of D-cycloserine uptake.

### Physiological fitness

Bacterial pathogens pay a physiological cost for the acquisition of resistance mutations. The physiological cost includes reduction of growth rate, less invasiveness or less transmissibility. However, the fitness cost of resistance mutations can be alleviated by fitness-compensatory mutations [71]. In this work, we found that UM 1072388579 carried a nsSNP in *rpoC* at the position of A734V. *rpoC* mutations have been reported as compensatory mutations which ameliorate the fitness costs incurred by mutations associated with rifampin resistance in both *Salmonella enterica* [72] and in *M*. *tuberculosis* [73]. These mutations have vital compensatory roles to increase mycobacterial transmissibility [73] and growth rate [74].

### Analysis of mutations related cell mobility, cell wall biogenesis pathway and transmembrane efflux pumps

**Fig 2:**
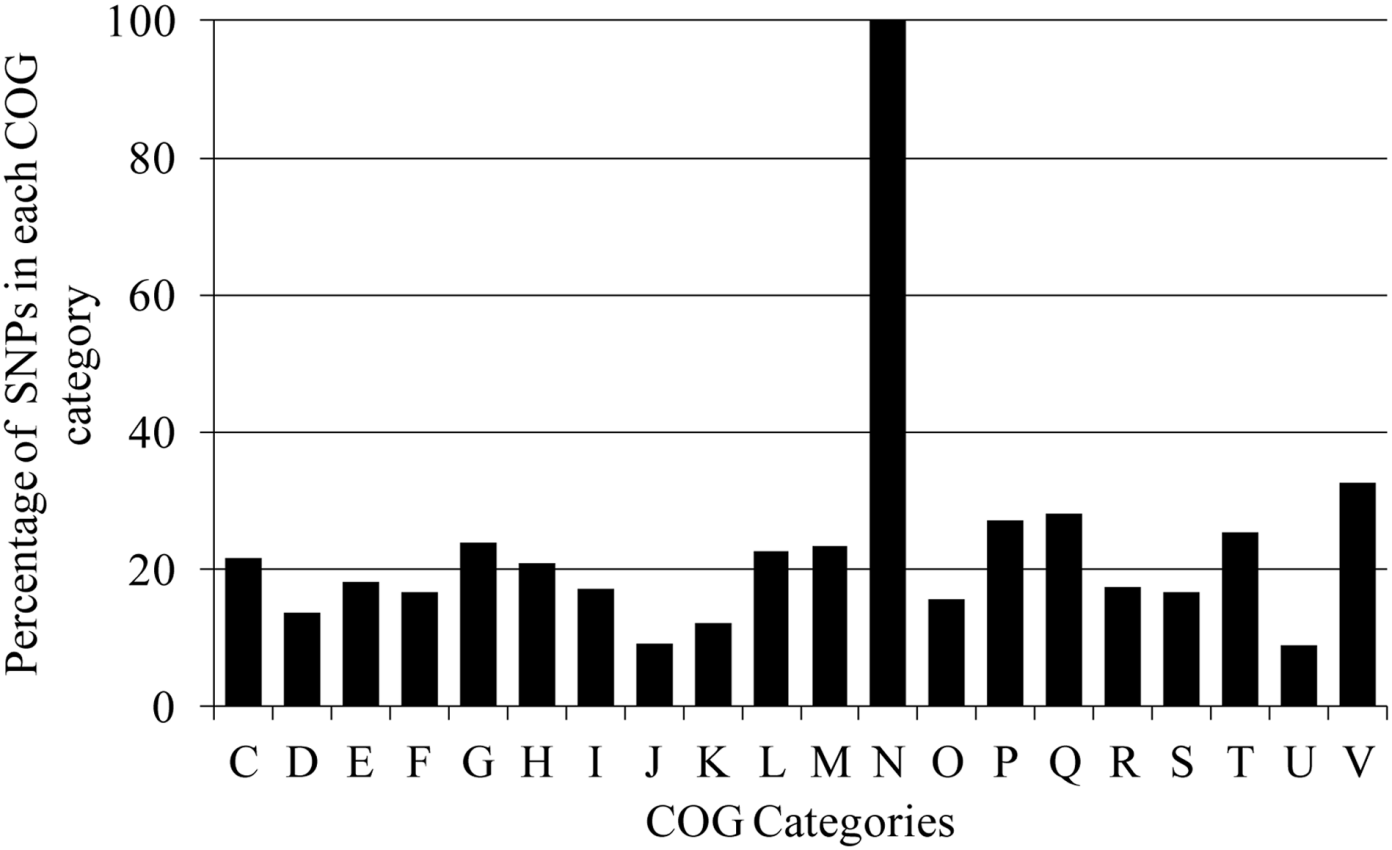
The distribution of individual genic SNPs into various functional groups based on COGs analysis. C: Energy production and conversion; D: Cell cycle control, cell division, chromosome partitioning; E: Amino acid transport and metabolism; F: Nucleotide transport and metabolism; G: Carbohydrate transport and metabolism; H: Coenzyme transport and metabolism; I: Lipid transport and metabolism; J: Translation, ribosomal structure and biogenesis; K: Transcription; L: Replication, recombination and repair; M: Cell wall/membrane/envelope biogenesis; N: Cell motility; O: Posttranslational modification, protein turnover, chaperones; P: Inorganic ion transport and metabolism; Q: Secondary metabolites biosynthesis, transport and catabolism; R: General function prediction only; S: Function unknown; T: Signal transduction mechanisms; U: Intracellular trafficking, secretion, and vesicular transport and V: Defense mechanisms.

For further functional analysis, all proteins with nsSNPs were classified into various functional groups based on COGs in order to thoroughly search for the gene SNPs that are involved in adaptation, transmission, survival as well as development of acquired drug resistance. The number of gene SNPs in each COG category was normalized with respect to the number of genes in the category and expressed as percentage (%). As reported in [16], UM 1072388579 contains much more missense mutations in proteins belonging to the category N (cell mobility) (Fig 2). Surprisingly, we found that all 65 genes encoding PE and PPE protein families (category N) underwent non-synonymous mutations. These two gene families occupy about 10% of the mycobacterial genome [37]. It has been extensively postulated that these protein families play important roles in bacterial virulence and evasion of antigen-specific host responses through antigenic variation [75].

Unlike most other pathogens, *M*. *tuberculosis* acquires drug resistance through the sequential acquisition and accumulation of resistance mutations in the chromosome during sub-optimal drug treatment instead of horizontal gene transfer [19]. Therefore, it is interesting to identify mutations in genes other than well-known drug-resistance genes. The reduction of drug-efflux pump activities and alteration of cell wall permeability provide a “stepping stone” for the development of full-blown antibiotics resistance [76]. In this regard, we mined the literature on the nsSNPs found in UM 1072388579 that are related to the cell wall biogenesis pathway and transmembrane drug-efflux pumps. The unusual complexity of the mycobacterial mycolic acid-containing cell wall renders a permeability barrier, making *M*. *tuberculosis* naturally resistant to many drugs [19]. With the knowledge of this intrinsic resistance mechanism, several anti-TB drugs, such as isoniazid [77] and ethambutol [78] were discovered to target the cell wall biogenesis pathways. Accumulating evidence revealed that mutations in the *mmpL*, *pks* and *fadD* genes are probably associated with drug resistance in *M*. *tuberculosis* [40, 55, 79]. There are 13 important *mmpL* genes in the *M*. *tuberculosis* genome, which encode membrane proteins that play an essential role in the lipids transport [80]. *pks* genes encoding the polyketide synthases are involved in the lipopolysaccharide and complex lipids biosynthesis. The co-localization of *mmpL* with *pks* and *fadD* in the *M*. *tuberculosis* genome proposes a function for these proteins in the transport of complex lipids [80]. A previous study further proved a functional crosstalk between pks13 and fadD32 enzymes in mycolic acid biosynthesis [81]. Mutations in the *mmpL*, *pks* and *fadD* genes were also suggested to have a compensatory role in drug resistance [40]. In this work, we identified quite a high density of mutations in these gene families (Table 5). However, there is no literature showing how mutations in these protein families can affect drug resistance in *M*. *tuberculosis*. We postulate that the mutations in these genes disrupt the cell wall structure and its permeability for anti-TB drugs.

**Table 5:** Summary of potential mutations associated with cell wall biogenesis pathway, transmembrane efflux pumps and transporters in UM 1072388579.

	Gene name	Mutation
**Cell wall biogenesis pathway**	*mmpL2*	R426H
	*mmpL5*	I948V and T794I
	*mmpL9*	H328P
	*mmpL12*	S381P
	*mmpL13a*	A276fs
	*pks3*	X489Y
	*pks5*	L2061R and L2061R
	*pks6*	E204A, R1402P and N28fs
	*pks7*	E814A, L973P and F1489C
	*pks8*	A1357T
	*pks12*	P3649A, H2147Q, R1652C, P236fs and V238fs
	*pks15*	V333A and G488fs
	*FadD2*	I81M
	*FadD11*.*1*	P5fs
	*FadD13*	T213A
	*FadD14*	E150G
	*FadD15*	T100I
	*FadD21*	L543I
	*FadD23*	E422Q
	*FadD29*	W19L
	*FadD30*	P207L
	*FadD32*	G227S
	*FadD34*	S16W
**Transmembrane efflux pumps and transporters**	Rv0194	M74T and P1098L
	Rv1218c	Q243P
	Rv1458c	T133A
	Rv2688	P156T

We then analyzed mutations in transmembrane efflux pumps and transporters which belong to the category V (defense mechanism) as they may be directly associated with the development of XDR phenotype. We found that UM 1072388579 carried several non-synonymous mutations in ATP-binding protein ABC transporters Rv0194 (M74T and P1098L), tetronasin-transport ATP-binding protein ABC transporter Rv1218c (Q243P), antibiotic-transport ATP-binding protein ABC transporter Rv1458c (T133A) and antibiotic-transport ATP-binding protein ABC transporter Rv2688 (P156T) (Table 5). As previously reported [82], overexpression of ATP-binding protein ABC transporters Rv0194 conferred multidrug (ampicillin, vancomycin, novobiocin, and erythromycin) resistance to *Mycobacterium smegmatis*. Resistant *M*. *tuberculosis* strains tend to contain more mutations in the ATP-binding protein ABC transporters Rv0194 compared to that of the sensitive strains [55]. Balganesh and collaborators (2010) demonstrated that the ΔRv1218c mutant of *M*. *tuberculosis* showed a significant increase in the inhibitory for ethidium bromide, bisanilopyrimidines (BAPS), pyrroles novobiocins, biarylpiperazines and pyridines compared to that of the wild-type *M*. *tuberculosis* [83]. This suggests that Rv1218c plays a role to efflux these compounds from *M*. *tuberculosis*. Moreover, the expression level of Rv1218c [84] and Rv1458c [85] are closely related to the formation of MDR and XDR phenotypes. Previous study further proved that Rv2686c-Rv2687c-Rv2688c proteins are active ABC drug transporters which pump out ciprofloxacin from the bacterial cell [86].

### Analysis of mutations in intergenic regions

Compared to the SNPs, the role of IGRs in the formation of drug resistance has received little attention. Gene promoters, part of the IGRs, are the regulatory regions that govern the expression of downstream genes [87]. A recent promoter analysis indicated that mutations in the *thyA* and *thyX* promoters would lead to the up-regulation of *thyA* and *thyX* expression [40]. We predicted 57 putative promoters containing SNPs (S4 Table). Of them, SNP −4 (C/T) in the proximal promoter region of the *TetR*/*acrR* gene is of interest. For convenient description hereafter, the nucleotide A at the ATG translational start site of the gene was designated +1, and nucleotides upstream of +1 were assigned negative numbers. AcrR is a transcriptional repressor which negatively regulates the AcrAB operon [88]. In *Escherichia coli*, mutations in *acrR* gene significantly increase the expression level of AcrA, a component of the AcrAB multi-drug efflux pump to confer a high-level resistance to fluoroquinolones [89]. Therefore, it is possible that the fluoroquinolones resistance in UM 1072388579 is contributed by the reduced cellular accumulation of fluoroquinolones by the AcrAB multi-drug efflux pump in addition to the alteration of drug targeted DNA gyrase.

We then further examined whether the other 48 *M*. *tuberculosis* strains contain this unique promoter SNP. Thorough analysis indicated that this mutation is present in 15 *M*. *tuberculosis* strains (including UM 1072388579). All belong to the East Asian lineage (Table 6 and S2 Fig). Among them, nine strains (UM_1072388579, FJ05194, X122, XDR1221, XDR1219, WX1, WX3, CTRI-4 and NZXDR1) exhibit the XDR phenotype. *M*. *tuberculosis* 210 and HN878 are drug susceptible strains, while G-12-005, OM-V02_005, R1207 and W-148 are known to be MDR *M*. *tuberculosis* strains. Although we cannot derive a conclusion that the presence of the mutation in the *TetR*/*acrR* promoter is correlated with drug resistance, the SNP may be an informative marker to recognize candidates of the East Asian lineage.

**Table 6:** Polymorphism of *TetR*/*acrR* promoter in *M*. *tuberculosis* strains.

No	Strain	Nucleotide at −4 allele	Genotype family^a^
1	H37Rv	C	Euro-American lineage 4 strain, T
2	43–16836	C	Indo-Oceanic lineage 1 strain
3	EAS054	C	Indo-Oceanic lineage 1 strain
4	MTB T17	C	Indo-Oceanic lineage 1 strain
5	MTB T46	C	Indo-Oceanic lineage 1 strain
6	MTB T92	C	Indo-Oceanic lineage 1 strain
7	OSDD271	C	Indo-Oceanic lineage 1 strain, EAI
8	CAS/NITR204	C	East African-Indian lineage 3
9	GuangZ0019	C	Euro-American lineage 4 strain, MANU2
10	98-R604 INH-RIF-EM	C	Euro-American lineage 4 strain, LAM family
11	C	C	Euro-American lineage 4 strain, X
12	C2	C	Euro-American lineage 4 strain, LAM family
13	CDC1551	C	Euro-American lineage 4 strain, X3
14	CTRI-2	C	Euro-American lineage 4 strain, LAM9
15	F11	C	Euro-American lineage 4 strain, LAM3
16	Harlem	C	Euro-American lineage 4 strain, Haarlem
17	INS_XDR	C	Euro-American lineage 4 strain, LAM
18	KZN_605	C	Euro-American lineage 4 strain, LAM4
19	KZN_R506	C	Euro-American lineage 4 strain, LAM4
20	KZN_V2475	C	Euro-American lineage 4 strain, LAM4
21	KZN_1435	C	Euro-American lineage 4 strain, LAM4
22	KZN_4207	C	Euro-American lineage 4 strain, LAM4
23	OSDD105	C	Euro-American lineage 4 strain, T2
24	OSDD493	C	Euro-American lineage 4 strain, Ural
25	OSDD515	C	Euro-American lineage 4 strain, Uganda 1
26	PanR0201	C	Euro-American lineage 4 strain, LAM-c2
27	PanR0411	C	Euro-American lineage 4 strain, LAM9-c1
28	PanR0610	C	Euro-American lineage 4 strain, LAM-c2
29	PanR0902	C	Euro-American lineage 4 strain, Haarlem
30	PanR0906	C	Euro-American lineage 4 strain, LAM-c3
31	MAF CPHL_A	C	*Mycobacterium africanum* (West Africa 1) lineage 5
32	MAF GM041182	C	*Mycobacterium africanum* (West Africa 2) lineage 6
33	MAF K85	C	*Mycobacterium africanum* (West Africa 2) lineage 6
35	PRO5	C	Indo-Oceanic lineage 1 strain
36	UM 1072388579	T	East Asia lineage 2 strain, non-Beijing
37	FJ05194	T	East Asia lineage 2 strain, non-Beijing
38	210	T	East-Asian lineage 2 strain, Beijing
39	CTRI-4	T	East-Asian lineage 2 strain, Beijing-like
40	G-12-005	T	East-Asian lineage 2 strain, Beijing
41	HN878	T	East-Asian lineage 2 strain, Beijing
42	NZXDR1	T	East-Asian lineage 2 strain
43	OM-V02_005	T	East-Asian lineage 2 strain, Beijing
44	R1207	T	East-Asian lineage 2 strain, Beijing
45	W-148	T	East-Asian lineage 2 strain, Beijing
46	WX1	T	East-Asian lineage 2 strain, Beijing
47	WX3	T	East-Asian lineage 2 strain, Beijing
48	X122	T	East-Asian lineage 2 strain, Beijing
49	XDR1219	T	East-Asian lineage 2 strain, Beijing
50	XDR1221	T	East-Asian lineage 2 strain, Beijing

^a^ LAM: Latin American Mediterranean; EAI: East African Indian

## Conclusions

In this work, we generated an improved UM 1072388579 genome using more advanced mate pair combined sequencing approach. Our analyses revealed that UM 1072388579 belongs to a non-Beijing clade of East Asian lineage 2 which is very close to the Beijing clade. Interestingly, we postulated that UM 1072388579 is an ancient strain, which most likely represents the ancestor of the Beijing clade. However, at this stage of knowledge, its evolutionary origin and history is only partially understood due to the limited genome sequences of such genotype family in Malaysia and other regions of the world. Genetic mutations related to first-line and second-line anti-TB drugs are well described. The presence of classical and uncommon SNPs in genes and IGRs allows our clinical isolate to escape from multiple tested drugs. Other SNPs listed in this study deserve specific attention as they may afford a selective advantage in the presence of antibiotic to adapt, survive and spread to its surrounding population. Further studies are required to confirm the functional importance of these mutations in the formation of XDR phenotype or greater fitness within the population. This report will help to evaluate the geographical and temporal dynamic of the occurrence of XDR-TB in Malaysia as well as other regions of the world. We hope that the faithful execution of UM 1072388579 genome sequence and in-depth analysis of the genome content will serve as a landmark for future TB research.

## Supporting Information

S1 Fig COG class annotation distribution of UM 1072388579 genome. A: RNA processing and modification; C: Energy production and conversion; D: Cell cycle control, cell division, chromosome partitioning; E: Amino acid transport and metabolism; F: Nucleotide transport and metabolism; G: Carbohydrate transport and metabolism; H: Coenzyme transport and metabolism; I: Lipid transport and metabolism; J: Translation, ribosomal structure and biogenesis; K: Transcription; L: Replication, recombination and repair; M: Cell wall/membrane/envelope biogenesis; N: Cell motility; O: Posttranslational modification, protein turnover, chaperones; P: Inorganic ion transport and metabolism; Q: Secondary metabolites biosynthesis, transport and catabolism; R: General function prediction only; S: Function unknown; T: Signal transduction mechanisms; U: Intracellular trafficking, secretion, and vesicular transport and V: Defense mechanisms.
(TIF)

S2 Fig Multiple promoter sequence alignment of UM 1072388579 with other 48 *M*. *tuberculosis* strains. The ATG start codon is shown in boldface and assigned as +1, and nucleotides upstream of +1 were denoted as negative numbers. The SNP is highlighted in box.
(PDF)

S1 Table Description of *M*. *tuberculosis* strains used in this study. (PDF)

S2 Table Functional annotation of genes in UM 1072388579 strain. Genes predicted in UM 1072388579 genome have been annotated by local BLAST similarity searches against NCBI nr, SwissProt and COG databases.
(XLSX)

S3 Table Single nucleotide polymorphisms identified in UM 1072388579. (XLSX)

S4 Table Non-coding regions whose predicted promoter regions contain SNPs. The flanking genes of promoters containing SNPs are functional annotated using local BLAST similarity searches against NCBI nr, SwissProt and COG databases.
(XLSX)
