# Supplementary figures and images for: Genome Analysis of the First Extensively Drug-Resistant (XDR) Mycobacterium tuberculosis in Malaysia Provides Insights into the Genetic Basis of Its Biology and Drug Resistance
Source: PLoS One. 2015 Jun 25;10(6):e0131694. doi: 10.1371/journal.pone.0131694 (PMC4481353; doi:10.1371/journal.pone.0131694)

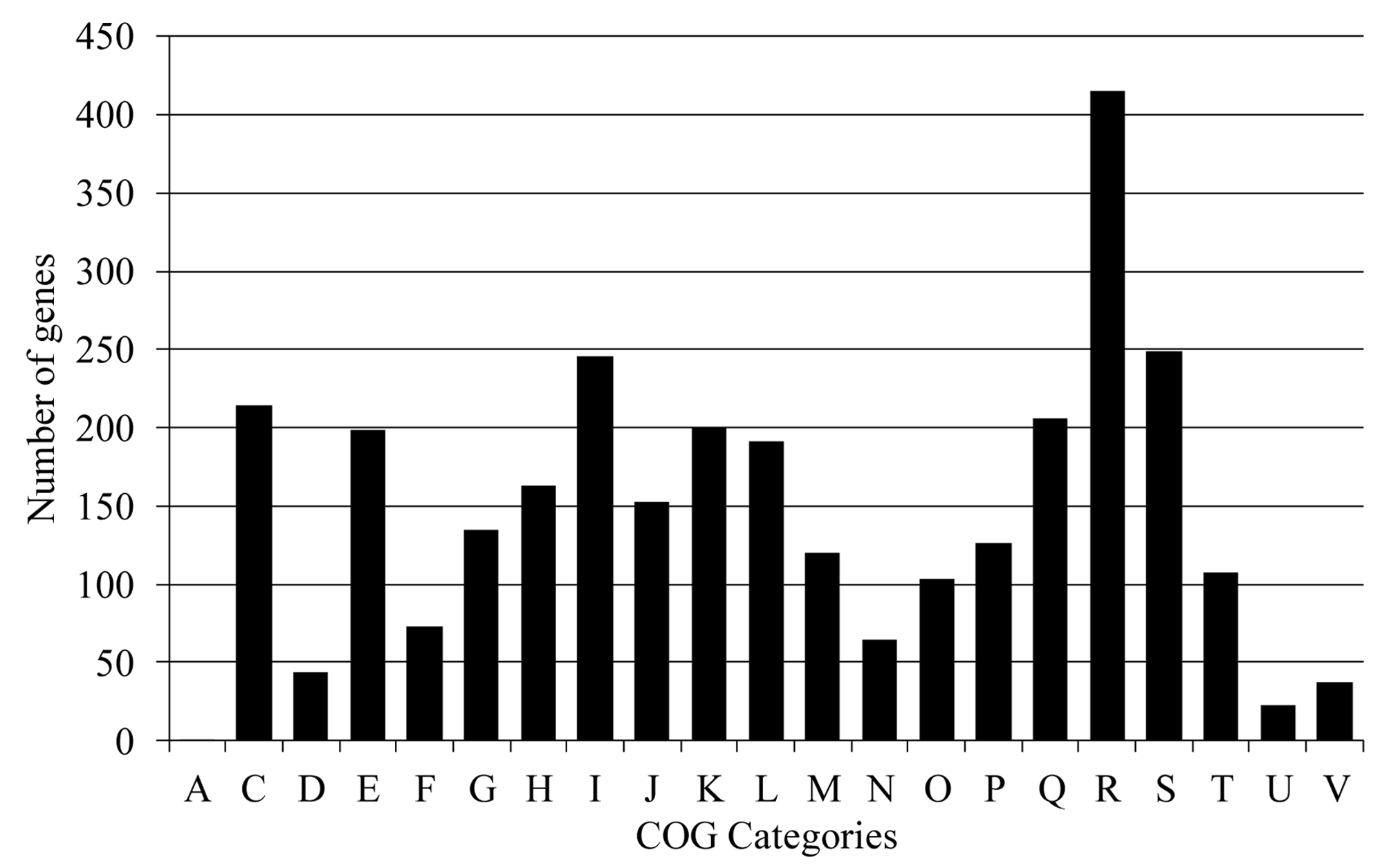

Supplement: S1 Fig — A: RNA processing and modification; C: Energy production and conversion; D: Cell cycle control, cell division, chromosome partitioning; E: Amino acid transport and metabolism; F: Nucleotide transport and metabolism; G: Carbohydrate transport and metabolism; H: Coenzyme transport and metabolism; I: Lipid transport and metabolism; J: Translation, ribosomal structure and biogenesis; K: Transcription; L: Replication, recombination and repair; M: Cell wall/membrane/envelope biogenesis; N: Cell motility; O: Posttranslational modification, protein turnover, chaperones; P: Inorganic ion transport and metabolism; Q: Secondary metabolites biosynthesis, transport and catabolism; R: General function prediction only; S: Function unknown; T: Signal transduction mechanisms; U: Intracellular trafficking, secretion, and vesicular transport and V: Defense mechanisms. (TIF) [file pone.0131694.s001.tif]
