# Supplementary material for: Genome Analysis of the First Extensively Drug-Resistant (XDR) Mycobacterium tuberculosis in Malaysia Provides Insights into the Genetic Basis of Its Biology and Drug Resistance
Source: PLoS One. 2015 Jun 25;10(6):e0131694. doi: 10.1371/journal.pone.0131694 (PMC4481353; doi:10.1371/journal.pone.0131694)
Supplement: S2 Fig — The ATG start codon is shown in boldface and assigned as +1, and nucleotides upstream of +1 were denoted as negative numbers. The SNP is highlighted in box. (PDF) [file pone.0131694.s002.pdf]

```
                                -39                                          +3
43-16836                   CACGCGCTAAGGGGTGTTGTAAACAGGTCGGTATACTCAGCACTATG
C                          CACGCGCTAAGGGGTGTTGTAAACAGGTCGGTATACTCAGCACTATT
98-R604_INH-RIF-EM         CACGCGCTAAGGGGTGTTGTAAACAGGTCGGTATACTCAGCACTATG
bovis                      CACGCGCTAAGGGGTGTTGTAAACAGGTCGGTATACTCAGCACTATG
CDC1551                    CACGCGCTAAGGGGTGTTGTAAACAGGTCGGTATACTCAGCACTATG
EAS054                     CACGCGCTAAGGGGTGTTGTAAACAGGTCGGTATACTCAGCACTATG
F11                        CACGCGCTAAGGGGTGTTGTAAACAGGTCGGTATACTCAGCACTATG
H37Rv                      CACGCGCTAAGGGGTGTTGTAAACAGGTCGGTATACTCAGCACTATG
Harlem                     CACGCGCTAAGGGGTGTTGTAAACAGGTCGGTATACTCAGCACTATG
INS_XDR                    CACGCGCTAAGGGGTGTTGTAAACAGGTCGGTATACTCAGCACTATG
KZN605                     CACGCGCTAAGGGGTGTTGTAAACAGGTCGGTATACTCAGCACTATG
KZN_R506                   CACGCGCTAAGGGGTGTTGTAAACAGGTCGGTATACTCAGCACTATG
KZN_V2475                  CACGCGCTAAGGGGTGTTGTAAACAGGTCGGTATACTCAGCACTATG
KZN-1435                   CACGCGCTAAGGGGTGTTGTAAACAGGTCGGTATACTCAGCACTATG
KZN-4207                   CACGCGCTAAGGGGTGTTGTAAACAGGTCGGTATACTCAGCACTATG
MAF_CPHL_A                 CACGCGCTAAGGGGTGTTGTAAACAGGTCGGTATACTCAGCACTATG
GM041182                   CACGCGCTAAGGGGTGTTGTAAACAGGTCGGTATACTCAGCACTATG
MAF_K85                    CACGCGCTAAGGGGTGTTGTAAACAGGTCGGTATACTCAGCACTATG
MTB_T17                    CACGCGCTAAGGGGTGTTGTAAACAGGTCGGTATACTCAGCACTATG
MTB_T46                    CACGCGCTAAGGGGTGTTGTAAACAGGTCGGTATACTCAGCACTATG
MTB_T92                    CACGCGCTAAGGGGTGTTGTAAACAGGTCGGTATACTCAGCACTATG
OSDD271                    CACGCGCTAAGGGGTGTTGTAAACAGGTCGGTATACTCAGCACTATG
OSDD493                    CACGCGCTAAGGGGTGTTGTAAACAGGTCGGTATACTCAGCACTATG
PanR0201                   CACGCGCTAAGGGGTGTTGTAAACAGGTCGGTATACTCAGCACTATG
PanR0610                   CACGCGCTAAGGGGTGTTGTAAACAGGTCGGTATACTCAGCACTATG
PanR0902                   CACGCGCTAAGGGGTGTTGTAAACAGGTCGGTATACTCAGCACTATG
PanR0906                   CACGCGCTAAGGGGTGTTGTAAACAGGTCGGTATACTCAGCACTATG
CTRI-2                     CACGCGCTAAGGGGTGTTGTAAACAGGTCGGTATACTCAGCACTATG
PanR0411                   CACGCGCTAAGGGGTGTTGTAAACAAGTCGGTATACTCAGCACTATG
210                        CACGCGCTAAGGGGTGTTGTAAACAGGTCGGTATACTCAGTACTATG
CTRI-4                     CACGCGCTAAGGGGTGTTGTAAACAGGTCGGTATACTCAGTACTATG
FJ05194                    CACGCGCTAAGGGGTGTTGTAAACAGGTCGGTATACTCAGTACTATG
G-12-005                   CACGCGCTAAGGGGTGTTGTAAACAGGTCGGTATACTCAGTACTATG
HN878                      CACGCGCTAAGGGGTGTTGTAAACAGGTCGGTATACTCAGTACTATG
NZXDR1                     CACGCGCTAAGGGGTGTTGTAAACAGGTCGGTATACTCAGTACTATG
R1207                      CACGCGCTAAGGGGTGTTGTAAACAGGTCGGTATACTCAGTACTATG
UM_1072388579              CACGCGCTAAGGGGTGTTGTAAACAGGTCGGTATACTCAGTACTATG
WX1                        CACGCGCTAAGGGGTGTTGTAAACAGGTCGGTATACTCAGTACTATG
X122                       CACGCGCTAAGGGGTGTTGTAAACAGGTCGGTATACTCAGTACTATG
OM-V02_005                 CACGCGCTAAGGGGTGTTGTAAACAGGTCGGTATACTCAGTACTATG
W-148                      CACGCGCTAAGGGGTGTTGTAAACAGGTCGGTATACTCAGTACTATG
WX3                        CACGCGCTAAGGGGTGTTGTAAACAGGTCGGTATACTCAGTACTATG
XDR1219                    CACGCGCTAAGGGGTGTTGTAAACAGGTCGGTATACTCAGTACTATG
XDR1221                    CACGCGCTAAGGGGTGTTGTAAACAGGTCGGTATACTCAGTACTATG
PR05                       CACGCGCTAAGGGGTGTTGTAAACAGGTCGGTATACTCAGCACTATG
OSDD515                    CACGCGCTAAGGGGTGTTGTAAACAGGTCGGTATACTCAGCACTATG
OSDD105                    CACGCGCTAAGGGGTGTTGTAAACAGGTCGGTATACTCAGCACTATG
GuangZ0019                 CACGCGCTAAGGGGTGTTGTAAACAGGTCGGTATACTCAGCACTATG
C2                         CACGCGCTAAGGGGTGTTGTAAACAGGTCGGTATACTCAGCACTATG
CAS_NITR204                CACGCGCTAAGGGGTGTTGTAAACAGGTCGGTATACTCAGCACTATG
                           ************************* ************** *****
```
