# Supplementary material for: Genome Analysis of the First Extensively Drug-Resistant (XDR) Mycobacterium tuberculosis in Malaysia Provides Insights into the Genetic Basis of Its Biology and Drug Resistance
Source: PLoS One. 2015 Jun 25;10(6):e0131694. doi: 10.1371/journal.pone.0131694 (PMC4481353; doi:10.1371/journal.pone.0131694)
Supplement: S1 Table — (PDF) [file pone.0131694.s003.pdf]

No	Species[1]	Strain (GenBank accession number)	Type[2]	Genotype family[3]	Geographic location	Reference
1	MTB	H37Rv (NC_000962)	DS	Euro-American lineage 4 strain, T		[1]
2	MTB	F11 (CP000717.1)	DS	Euro-American lineage 4 strain, LAM3	Western Cape of South Africa	[2]
3	MTB	PRO5 (AOMG00000000)	DS	Indo-Oceanic lineage 1 strain	Malaysia	[3]
4	MTB	43-16836 (ATNF00000000)	DS	Indo-Oceanic lineage 1 strain	Thailand	[4]
5	MTB	CTRI-2 (CP002992)	DS	Euro-American lineage 4 strain, LAM9	Vladimir, Russia (Central region)	[5]
6	MTB	C2 (JHAD00000000)	DS	Euro-American lineage 4 strain, LAM family	Kaohsiung, Taiwan	[6]
7	MTB	KZN_4207 (CP001662)	DS	Euro-American lineage 4 strain, LAM4	KwaZulu-Natal, South Africa	[7]
8	MTB	98-R604 INH-RIF-EM (ABVM00000000)	DS	Euro-American lineage 4 strain, LAM family	Not reported	-
9	MTB	C (AAKR01000000)	DS	Euro-American lineage 4 strain, X	New York, USA	[8]
10	MTB	210 (ADAB00000000)	DS	East-Asian lineage 2 strain, Beijing	Los Angeles, USA	
11	MTB	HN878 (ADNF00000000)	DS	East-Asian lineage 2 strain, Beijing	USA	[9]
12	MTB	CDC1551 (AE000516.2)	DS	Euro-American lineage 4 strain, X3	USA	
13	MTB	G-12-005 (JHUF00000000)	MDR	East-Asian lineage 2 strain, Beijing	Georgia	[10]
14	MTB	OSDD271 (AQQC00000000)	MDR	Indo-Oceanic lineage 1 strain, EAI	India	[11]
15	MTB	OSDD515 (AUXC01000000)	MDR	Euro-American lineage 4 strain, Uganda 1	India	[12]
16	MTB	OSDD105 (AUXD00000000)	MDR	Euro-American lineage 4 strain, T2	India	[13]
17	MTB	KZN_1435 (CP001658.1)	MDR	Euro-American lineage 4 strain, LAM4	KwaZulu-Natal, South Africa	[5]
18	MTB	KZN_V2475 (ACVT00000000)	MDR	Euro-American lineage 4 strain, LAM4	KwaZulu-Natal, South Africa	[7]
19	MTB	R1207 (ADNH00000000.1)	MDR	East-Asian lineage 2 strain, Beijing	Western Cape of South Africa	[9]
20	MTB	OM-V02_005 (BARZ00000000)	MDR	East-Asian lineage 2 strain, Beijing	Japan	[14]
21	MTB	W-148 (ACSX00000000.1)	MDR	East-Asian lineage 2 strain, Beijing	Rusia	-
22	MTB	WX1 (AJGM01000000)	MDR	East-Asian lineage 2 strain, Beijing	WuXi,China	[15]
23	MTB	WX3 (AJGL01000000)	MDR	East-Asian lineage 2 strain, Beijing	WuXi,China	[15]
24	MTB	Harlem (AASN00000000)	MDR	Euro-American lineage 4 strain, Haarlem	The Netherlands	-
25	MTB	PanR0902 (ATSK00000000)	MDR	Euro-American lineage 4 strain, Haarlem	Panama	[16]
26	MTB	PanR0411 (ATDY00000000)	MDR	Euro-American lineage 4 strain, LAM9-c1	Panama	[16]

[1]MTB: *Mycobacterium tuberculosis*
[2]DS: drug susceptible; MDR: multidrug resistant; XDR: extensively-drug resistant
[3]LAM: Latin American Mediterranean; EAI: East African Indian

No	Species[1]	Strain (GenBank accession number)	Type[2]	Genotype family[3]	Geographic location	Reference
27	MTB	PanR0906 (ATSN00000000)	MDR	Euro-American lineage 4 strain, LAM-c3	Panama	[16]
28	MTB	PanR0610 (ATSA00000000)	MDR	Euro-American lineage 4 strain, LAM-c2	Panama	[16]
29	MTB	PanR0201 (ANZG00000000)	MDR	Euro-American lineage 4 strain, LAM-c2	Panama	[16]
30	MTB	UM 1072388579 (AMXW00000000)	XDR	East Asia lineage 2 strain, non-Beijing	Kuala Lumpur, Malaysia	This study
31	MTB	INS_XDR (JANH00000000)	XDR	Euro-American lineage 4 strain, LAM family	Peru	[17]
32	MTB	NZXDR1 (CCBK000000000)	XDR	East Asia lineage 2 strain	New Zealand	[18]
33	MTB	KZN_605 (ABGN00000000)	XDR	Euro-American lineage 4 strain, LAM4	KwaZulu-Natal, South Africa	-
34	MTB	OSDD493 (AVQJ00000000)	XDR	Euro-American lineage 4 strain, Ural	India	[19]
35	MTB	CTRI-4 (AIIE01000000)	XDR	East-Asian lineage 2 strain, Beijing-like	Tomsk, Rusia (Siberia)	[5]
36	MTB	KZN_R506 (ACVU00000000)	XDR	Euro-American lineage 4 strain, LAM4	KwaZulu-Natal, South Africa	[7]
37	MTB	GuangZ0019 (ANFI01000001)	XDR	Euro-American lineage 4 strain, MANU2	Guangdong, China	[20]
38	MTB	FJ05194 (ANBL01000000)	XDR	East Asia lineage 2 strain, non-Beijing	Fujian, China	[20]
39	MTB	XDR1219 (AJGO00000000.1)	XDR	East-Asian lineage 2 strain, Beijing	Shanghai, China	[15]
40	MTB	XDR1221 (AJGN01000000)	XDR	East-Asian lineage 2 strain, Beijing	Shanghai, China	[15]
41	MTB	PanR0411 (ATDY00000000)	MDR	Euro-American lineage 4 strain, LAM-c1	Panama	[16]
42	MTB	X122 (ADNG00000000.1)	Pre-XDR	East-Asian lineage 2 strain, Beijing	Western Cape of South Africa	[9]
43	MTB	MTB T92	Unknown	Indo-Oceanic lineage 1 strain	The Philippines	[21]
44	MTB	MTB T17	Unknown	Indo-Oceanic lineage 1 strain	The Philippines	[21]
45	MTB	EAS054	Unknown	Indo-Oceanic lineage 1 strain	India	[22]
46	MTB	MTB T46	Unknown	Indo-Oceanic lineage 1 strain	The Philippines	[22]
47	MTB	CAS/NITR204	Unknown	East African-Indian lineage 3	Chennai, India	[22]
48	MAF	MAF CPHL_A	Unknown	Mycobacterium africanum (West Africa 1) lineage 5	California, USA	[22]
49	MAF	MAF GM041182 (FR878060)	Unknown	Mycobacterium africanum (West Africa 2) lineage 6	The Gambia	[22]
50	MAF	MAF K85	Unknown	Mycobacterium africanum (West Africa 2) lineage 6	Netherlands	[22]
51	MBV	BCG str. ATCC 35743	-	-	-	-

[1]MTB: *Mycobacterium tuberculosis*; MAF: *Mycobacterium africanum*

[2]DS: drug susceptible; MDR: multidrug resistant; XDR: extensively-drug resistant

[3]LAM: Latin American Mediterranean; EAI: East African Indian

Supplementary reference

1. Cole ST, Brosch R, Parkhill J, Garnier T, Churcher C, Harris D, Gordon SV, Eiglmeier K, Gas S, Barry CE, 3rd *et al*: **Deciphering the biology of *Mycobacterium tuberculosis* from the complete genome sequence**. *Nature* 1998, **393**(6685):537-544.

2. Gagneux S, Small PM: **Global phylogeography of *Mycobacterium tuberculosis* and implications for tuberculosis product development**. *Lancet Infect Dis* 2007, **7**(5):328-337.

3. Ismail A, Teh LK, Ngeow YF, Norazmi MN, Zainul ZF, Tang TH, Najimudin N, Salleh MZ: **Draft Genome Sequence of a Clinical Isolate of *Mycobacterium tuberculosis* Strain PR05**. *Genome Announc* 2013, **1**(3).

4. Viratyosin W, Kulawonganunchai S, Smittipat N, Juthayothin T, Penpassakarn P, Pasomsub E, Chantratita W, Chaiprasert A, Palittapongarnpim P: **Draft Genome Sequence of the *Mycobacterium tuberculosis* Strain 43-16836, Belonging to the Indo-Oceanic Lineage, Isolated From Tuberculous Meningitis in Thailand**. *Genome Announc* 2013, **1**(5).

5. Ilina EN, Shitikov EA, Ikryannikova LN, Alekseev DG, Kamashev DE, Malakhova MV, Parfenova TV, Afanas'ev MV, Ischenko DS, Bazaleev NA *et al*: **Comparative genomic analysis of *Mycobacterium tuberculosis* drug resistant strains from Russia**. *PLoS One* 2013, **8**(2):e56577.

6. Liao YC, Chen YY, Lin HH, Chang JR, Su IJ, Huang TS, Dou HY: **Draft Genome Sequence of the *Mycobacterium tuberculosis* Clinical Isolate C2, Belonging to the Latin American-Mediterranean Family**. *Genome Announc* 2014, **2**(3).

7. Ioerger TR, Koo S, No EG, Chen X, Larsen MH, Jacobs WR, Jr., Pillay M, Sturm AW, Sacchettini JC: **Genome analysis of multi- and extensively-drug-resistant tuberculosis from KwaZulu-Natal, South Africa**. *PLoS One* 2009, **4**(11):e7778.

8. Friedman CR, Quinn GC, Kreiswirth BN, Perlman DC, Salomon N, Schluger N, Lutfey M, Berger J, Poltoratskaia N, Riley LW: **Widespread dissemination of a drug-susceptible strain of *Mycobacterium tuberculosis***. *J Infect Dis* 1997, **176**(2):478-484.

9. Ioerger TR, Feng Y, Chen X, Dobos KM, Victor TC, Streicher EM, Warren RM, Gey van Pittius NC, Van Helden PD, Sacchettini JC: **The non-clonality of drug resistance in Beijing-genotype isolates of Mycobacterium tuberculosis from the Western Cape of South Africa**. *BMC Genomics* 2010, **11**:670.

10. Berland JL, de Carvalho FM, de Almeida LG, Bablishvili N, Gauthier M, Paranhos-Baccala G, de Vasconcelos AT: **Draft Genome Sequence of *Mycobacterium tuberculosis* Clinical Strain G-12-005**. *Genome Announc* 2014, **2**(3).

11. Karuthedath Vellarikkal S, Patowary A, Singh M, Periwal V, Singh AV, Singh PK, Garg P, Mohan Katoch V, Katoch K, Jangir PK *et al*: **Draft Genome Sequence of a Clinical Isolate of Multidrug-Resistant *Mycobacterium tuberculosis* East African Indian Strain OSDD271**. *Genome Announc* 2013, **1**(4).

12. Vellarikkal SK, Singh AV, Singh PK, Garg P, Katoch VM, Katoch K, Chauhan DS, Sivasubbu S, Scaria V: **Draft Genome Sequence of Multidrug-Resistant *Mycobacterium tuberculosis* Clinical Isolate OSDD515, Belonging to the Uganda I Genotype**. *Genome Announc* 2013, **1**(6).

13. Vellarikkal SK, Singh AV, Singh PK, Garg P, Katoch VM, Katoch K, Chauhan DS, Sivasubbu S, Scaria V: **Draft Genome Sequence of a Multidrug-Resistant Clinical Isolate of *Mycobacterium tuberculosis* Belonging to a Novel Spoligotype**. *Genome Announc* 2013, **1**(6).

14. Tateishi Y, Tamaru A, Ogura Y, Niki M, Wada T, Yamamoto T, Hirata K, Hayashi T, Matsumoto S: **Whole-Genome Sequence of the Potentially Hypertransmissible Multidrug-Resistant *Mycobacterium tuberculosis* Beijing Strain OM-V02_005**. *Genome Announc* 2013, **1**(4).

15. Wu W, Zheng H, Zhang L, Wen Z, Zhang S, Pei H, Yu G, Zhu Y, Cui Z, Hu Z *et al*: **A genome-wide analysis of multidrug-resistant and extensively drug-resistant strains of *Mycobacterium tuberculosis* Beijing genotype**. *Mol Genet Genomics* 2013, **288**(9):425-436.

16. Lanzas F, Karakousis PC, Sacchettini JC, Ioerger TR: **Multidrug-resistant tuberculosis in panama is driven by clonal expansion of a multidrug-resistant *Mycobacterium tuberculosis* strain related to the KZN extensively drug-resistant *M. tuberculosis* strain from South Africa**. *J Clin Microbiol* 2013, **51**(10):3277-3285.

17. Guio H, Tarazona D, Galarza M, Borda V, Curitomay R: **Genome analysis of 17 extensively drug-resistant strains reveals new potential mutations for resistance**. *Genome Announc* 2014, **2**(4).

18. O'Toole RF, Johari BM, Mac Aogain M, Rogers TR, Bower JE, Basu I, Freeman JT: **Draft Genome Sequence of the First Isolate of Extensively Drug-Resistant *Mycobacterium tuberculosis* in New Zealand**. *Genome Announc* 2014, **2**(3).

19. Karuthedath Vellarikkal S, Vir Singh A, Kumar Singh P, Garg P, Mohan Katoch V, Katoch K, Chauhan DS, Scaria V, Sivasubbu S: **Draft Genome Sequence of an Extensively Drug-Resistant *Mycobacterium tuberculosis* Clinical Isolate of the Ural Strain OSDD493**. *Genome Announc* 2013, **1**(6).

20. Lin N, Liu Z, Zhou J, Wang S, Fleming J: **Draft Genome Sequences of Two Super-XDR Isolates of *M. tuberculosis* from China**. *FEMS Microbiol Lett* 2013.

21. Comas I, Chakravartti J, Small PM, Galagan J, Niemann S, Kremer K, Ernst JD, Gagneux S: **Human T cell epitopes of *Mycobacterium tuberculosis* are evolutionarily hyperconserved**. *Nat Genet* 2010, **42**(6):498-503.

22. Broad Institute website. [www.broadinstitute.org/]
